# Thermal tolerance and heritability in dune-dwelling ants reveal bioindicator potential for climate vulnerability in coastal ecosystems

**DOI:** 10.1007/s00484-025-03081-5

**Published:** 2026-02-06

**Authors:** Karollina Vieira da Conceição, Maykon Passos Cristiano, Danon Clemes Cardoso

**Affiliations:** 1 Departamento de Biodiversidade Evolução e Meio Ambiente, Universidade Federal de Ouro Preto, Ouro Preto, MG Brazil; 2 Grupo de Pesquisa em Genética e Evolução de Formigas, Universidade Federal de Ouro Preto, Mina Gerais Ouro Preto, Brazil

**Keywords:** Thermal tolerance, Heritability, Chronobiology, Coastal zones, Ectotherms, Dune ants, Conservation physiology

## Abstract

Climate change is expected to intensify thermal stress in coastal ecosystems, threatening biodiversity and ecosystem functioning. In this study, we investigate species-specific and colony-level variation in thermal tolerance among three psammophilous ant species (*Mycetophylax* spp.) inhabiting Brazilian coastal dunes. Using critical thermal limits (CTmin and CTmax), linear mixed-effects models, and heritability estimates, we assessed the role of diel activity rhythms and genetic structure in shaping thermal performance. Results revealed that *M. simplex*, a nocturnal and substrate-specialized species, exhibited significantly lower CTmin and CTmax values compared to diurnal congeners, and that colony identity explained a substantial portion of variance (H² = 0.53 for CTmin, H² = 0.39 for CTmax). These findings suggest limited thermal resilience and evolutionary constraints in *M. simplex*, reinforcing its potential as a bioindicator of thermal vulnerability. Given projected warming and habitat disturbance in southeastern Brazil, we highlight the importance of integrating functional traits and genetic metrics into environmental monitoring and conservation planning.

**Supplementary Information:**

The online version contains supplementary material available at 10.1007/s00484-025-03081-5.

## Introduction

Thermal stress is one of the most pervasive and biologically disruptive consequences of climate change, particularly in tropical and coastal ecosystems where many species already operate near their physiological thermal limits (Bennett et al. 2021). As temperature governs the metabolic, ecological, and evolutionary dynamics of ectothermic organisms (Angilletta 2009; Chown and Nicolson 2004), even small shifts in thermal regimes can reshape ecological interactions and compromise population viability. Insects, and ants in particular, are especially vulnerable to thermal stress due to their dependence on external temperatures to regulate physiological performance (Roeder et al. 2021). Because ectotherms lack efficient internal thermoregulation, they rely on behavioral and physiological adjustments to cope with environmental fluctuations. The critical thermal limits, such as the critical thermal minimum (CTmin) and maximum (CTmax), have emerged as key functional traits for evaluating species vulnerability to warming, environmental degradation, and extreme events. These traits determine the boundaries of ecological performance and inform species distribution patterns, sensitivity to disturbance, and adaptive potential (Bishop et al. 2017; Bennett et al. 2021; Câmara et al. 2024). Thus, understanding how CTmin and CTmax vary across species and environments is critical for predicting biological responses to global warming and guiding conservation strategies based on functional traits.

Different predictors have been proposed to be related to critical thermal limits. While body size has been proposed as a predictor of thermal limits, its effects are inconsistent across ecological contexts (Clusella-Trullas et al. 2011; Roeder et al. 2021). In ants, thermal tolerance is modulated by multiple interacting factors, including seasonality (Bujan et al. 2020a, b), nutritional state (Bujan and Kaspari 2017), diel foraging rhythms (Garcia-Robledo et al. 2017), and microhabitat structure (Verble-Pearson et al. 2015). Diurnally active species typically exhibit higher CTmax values, reflecting acclimatory or plastic responses to elevated temperatures (Kaspari et al. 2016).

Although CTmax has become a widely used measure in thermal biology, questions have been raised about how precisely it can be determined, especially regarding the influence of heating rates on the outcome (Lutterschmidt and Hutchison 1997; Terblanche et al. 2007; Leong et al. 2022). Nevertheless, CTmax remains a widely adopted and informative metric, offering valuable insights into species’ thermal limits, particularly when interpreted within robust experimental and conceptual frameworks (Ørsted et al. 2022). Still, the evolutionary and ecological drivers of CTmax remain understudied, especially in vulnerable tropical regions. This knowledge gap is particularly relevant in dynamic and fragile ecosystems such as Brazil restinga, which faces growing threats from climate change, habitat fragmentation, and coastal development. In these contexts, understanding not only the range of thermal tolerance but also the heritability of such traits is essential to assess species long-term potential for persistence and adaptation (Diamond et al. 2012; Câmara et al. 2024).

Trait-based approaches that incorporate genetic structure, physiological limits, and behavioral strategies offer a powerful framework for evaluating climate vulnerability and identifying bioindicator species. The genus *Mycetophylax* (Myrmicinae: Attini: Attina) represents a compelling model system in this regard. These fungus-farming ants are endemic to coastal sand dunes of South America and exhibit marked differences in microhabitat use, foraging behavior, and geographical distribution (Cardoso et al. 2012, 2014, 2015, 2021). Given their narrow ecological niches and exposure to thermal extremes, *Mycetophylax* species may function as effective indicators of environmental stress in coastal landscapes.

In this study, we investigate both intra- and interspecific variation in thermal tolerance among *Mycetophylax* species by combining standardized thermal assays with linear mixed-effects models. We assess the influence of species identity, diel activity patterns, and colony structure on CTmin and CTmax, and estimate the heritability of these traits to evaluate their potential for evolutionary response. Our findings provide mechanistic insights into how environmental filtering, behavioral specialization, and genetic constraints interact to shape species’ vulnerability to thermal stress. These results have direct implications for monitoring and conserving thermally sensitive organisms in coastal ecosystems increasingly affected by climate change.

## Model species

The ant genus *Mycetophylax* (Formicidae: Myrmicinae: Attini: Attina) comprises an early-diverging lineage of fungus-farming ants inhabiting coastal sand dune ecosystems, characterized by microhabitat specialization, behavioral divergence, and close phylogenetic relatedness. These features make *Mycetophylax* an ideal system for investigating how thermal tolerance is shaped by ecological filtering, activity patterns, and potential genetic constraints in coastal environments under climate stress.

To address these questions, we focus on three closely related psammophilous species: *Mycetophylax morschi*, *M. conformis*, and *M. simplex* that are monogynous, ground-nesting ants adapted to sandy dune environments. These species cultivate lower-attine fungi in subterranean chambers and are strongly influenced by soil texture and local microclimatic conditions (Klingenberg and Brandão 2009; Cardoso et al. 2021). Their geographic distributions are distinct yet partially overlapping: *Mycetophylax simplex* occurs along the southern coast (from Rio Grande do Sul to southern São Paulo), *M. conformis* spans from southeastern to northeastern Brazil, and *M. morschi* has the broadest range, including sympatric zones with both congeners along much of the Brazilian coastline. The dunes of Cabo Frio (Rio de Janeiro) represent the only known locality where all three species still co-occur (Cardoso et al. 2012, 2014, 2021).

These species also differ in diel foraging behavior. The *Mycetophylax simplex* is primarily nocturnal, initiating foraging at dusk and ceasing activity before sunrise, likely an adaptation to avoid desiccation and thermal stress (Diehl-Fleig and Diehl 2007). In contrast, *M. conformis* and *M. morschi* are diurnal, with activity concentrated in the early to mid-morning, when temperatures are moderate and humidity relatively high (Klingenberg et al. 2007; Cardoso et al. 2021). These patterns may reflect both resource partitioning and species-specific thermal tolerances.

Together, the behavioral, morphological, and ecological differences among these *Mycetophylax* species provide a robust framework for exploring how environmental and evolutionary forces shape thermal physiology. We hypothesize that CTmax values will be higher in the diurnal species, which are more frequently exposed to elevated surface temperatures, while *M. simplex* will exhibit lower CTmax due to nocturnal foraging. Conversely, we expect *M. simplex* to show lower CTmin values, reflecting enhanced tolerance to cooler nighttime conditions. We expect that colony identity will significantly explain variation in thermal tolerance, indicating a genetic basis for intercolonial differences and supporting the existence of evolutionary constraints on CTmin and CTmax. We further expect heritability to differ between CTmin and CTmax, given potential asymmetries in evolutionary constraints on cold versus heat tolerance.

## Materials and methods

### Thermal tolerance assays (CTmin and CTmax)

We assessed critical thermal minimum (CTmin) and maximum (CTmax) for worker ants of three psammophilous species—*Mycetophylax morschi*, *M. conformis*, and *M. simplex*—collected from coastal dune and restinga habitats in southeastern and southern Brazil. Colonies were transported to the laboratory and maintained for 24–48 h underneath controlled conditions (25 ± 1 °C, 12:12 h light–dark cycle, ad libitum access to water and food) prior to experimentation (see Cardoso et al. 2011). We randomly selected adult workers from multiple colonies per species for the thermal tolerance trials. To ensure precise control over temperature ramping, we used a programmable thermocycler (Thermo Fisher MiniAmp Thermal Cycler) to conduct dynamic assays. Preliminary calibration tests confirmed that the internal temperatures within PCR tubes closely matched the programmed block temperatures of the thermocycler (± 0.2 °C), ensuring accurate and consistent thermal exposure across trials.

Individual ants were placed in 0.5 mL PCR tubes, and thermal limits were assessed using a constant ramping rate of 0.25 °C per minute. For CTmax trials, the temperature increased from an initial 25 °C until the ant lost motor coordination, which was recorded as the critical thermal maximum. For CTmin, temperature decreased from 25 °C until the ant entered a chill coma, defined as the point at which it ceased all movement, even when gently stimulated. Each individual was tested only once and allowed a 10-minute acclimation at 25 °C within the thermocycler block before ramping began. We tested 15–30 individuals per species per trait, with individuals nested within colony ID. All trials were conducted in the same laboratory environment to control for humidity and light. A total of 368 individuals were tested, distributed across 23 colonies and classified by species and foraging activity type: diurnal or dusk-dawn. Because diel activity patterns are species-specific in our study system—with *M. simplex* foraging exclusively at night and *M. conformis* and *M. morschi* primarily during the day—there is a natural association between species identity and activity period. While this does not represent a strict confounding, it may limit our ability to fully disentangle their independent effects on thermal tolerance.

### Statistical analysis

We analyzed the thermal tolerance data using linear mixed-effects models (LMMs) to evaluate variation in critical thermal minimum (CTmin) and maximum (CTmax) across species and colonies. The response variable was the temperature at which each individual reached its thermal limit. Fixed effects included species identity and circadian rhythms, while colony identity was included as a random intercept to account for repeated measures within colonies.

All analyses were conducted in R version 4.2 (R Core Team 2023) using the RStudio environment. We used the lme4 (Bates et al. 2015) to fit mixed-effects models, and the lmerTest package (Kuznetsova et al. 2017) to obtain significance tests for fixed and random effects. Type II Wald chi-square tests were conducted using the Anova function from the car package (Fox and Weisberg 2019). Random effects were tested via likelihood ratio tests using the ranova function from lmerTest. To check model assumptions and assess residual patterns, we used the DHARMa package (Hartig 2022), which simulates residuals for diagnostic plotting. 

For each response variable, we first fitted full models including species, treatment type, and the random effect of colony (temp ~ species + cincardian + (1 | colony)). We also fitted the reduced model (temp ~ species + (1|colony)). Post hoc comparisons were not required, as our primary objective was to evaluate the effect of species on thermal tolerance rather than pairwise contrasts.

To estimate the broad-sense heritability (H²) of thermal tolerance traits, we fitted linear mixed-effects models separately for CTmax and CTmin using colony identity as a random effect. The models were implemented using the lme4 structure via the statsmodels package in Python. Each model included temperature (CTmax or CTmin) as the response variable and a random intercept for colony, following the structure: temp ~ 1 + (1|colony). Variance components were extracted from the fitted models to calculate H² as the proportion of phenotypic variance attributable to between-colony variation:

$$\:{H}^{2}=\frac{{{\upsigma\:}}_{colony}^{2}}{{{\upsigma\:}}_{colony}^{2}+{{\upsigma\:}}_{residual}^{2}}$$

 Where σ^2^_colony_ is the variance explained by colony identity and σ^2^_residual_ is the residual (within-colony) variance. Likelihood ratio tests (LRT) were used to evaluate whether including colony as a random effect significantly improved model fit compared to a null model with only an intercept.

## Results

**Fig. 1:**
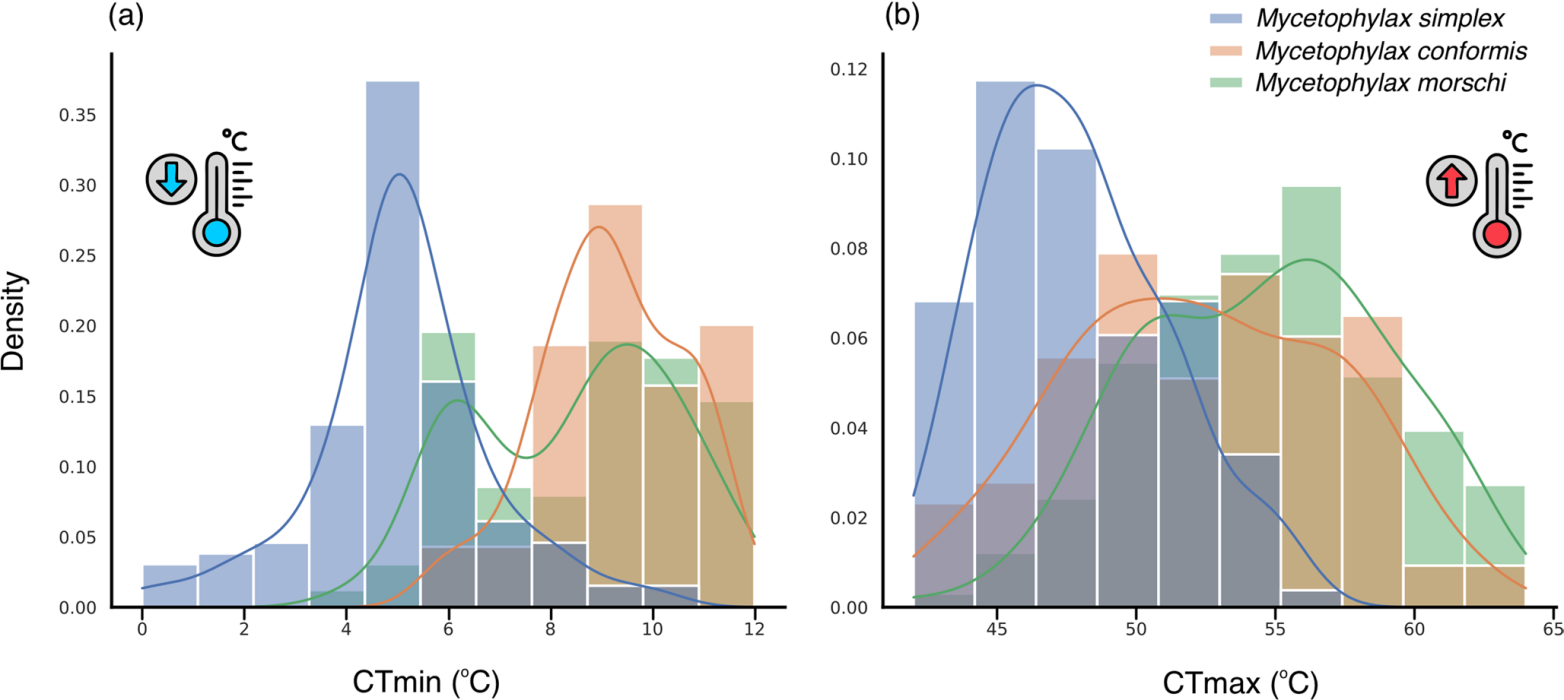
Distribution of critical thermal minimum (CTmin) and maximum (CTmax) temperatures recorded for individuals of psammophilous *Mycetophylax* species. Histograms with density curves illustrate differences in thermal tolerance across species and activity rhythms. *M. simplex* shows lower CTmin and CTmax values relative to *M. morschi* and *M. conformis*, highlighting species-specific physiological profiles aligned with diel foraging behavior

Thermal tolerance variation among *Mycetophylax* species is summarized in supplementary material table S1 and Fig. 1. *M. conformis* exhibited the highest mean CTmin and CTmax values, with CTmax ranging from 43 to 62 °C. *M. morschi* displayed a broad thermal range, with CTmin values from 4 to 12 °C and CTmax from 42 to 64 °C. In contrast, *M. simplex* had the lowest thermal limits among the three species. These patterns highlight clear interspecific differences in both cold and heat tolerance, with *M. simplex* exhibiting greater cold tolerance but reduced heat tolerance, as initially hypothesized. Therefore, dusk-dawn individuals of *M. simplex* showed significantly lower thermal thresholds: CTmin values were, on average, 3.57 °C lower, and CTmax values were 6.03 °C lower compared to diurnal individuals (*M. conformis* and *M. morschi*), indicating the influence of chronobiological factors on thermal performance.

**Fig. 2:**
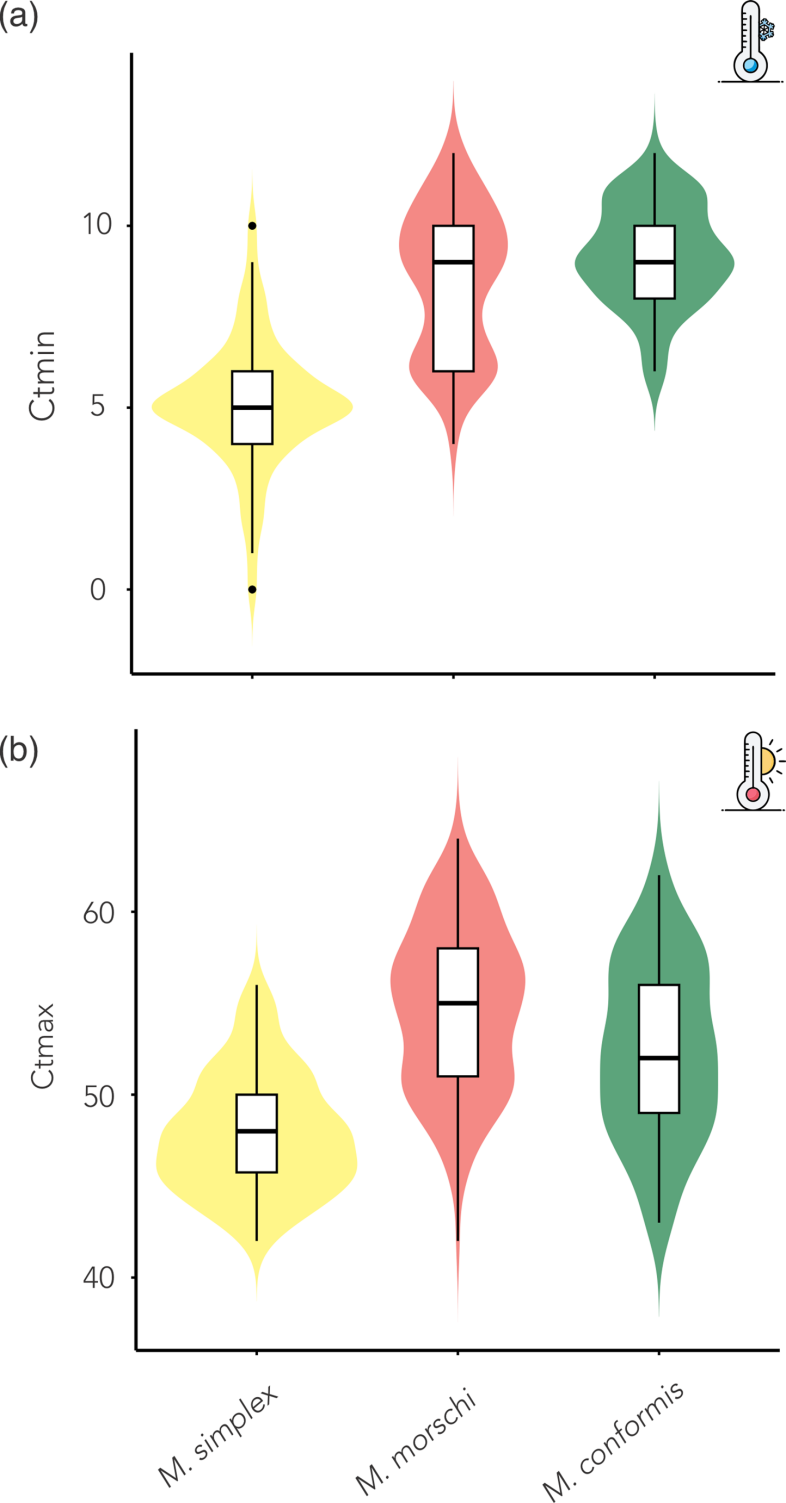
Violin plots of species-specific variation in critical thermal limits (CTmin and CTmax) among *Mycetophylax* ants. *M. simplex* exhibited significantly lower CTmin and CTmax values compared to the diurnal species *M. morschi* and *M. conformis* (CTmin: χ² = 143.94, *p* < 2.2 × 10⁻¹⁶; CTmax: χ² = 69.72, *p* < 7.2 × 10⁻¹⁶). Colony-level variance was significant in both models (LRT_CTmin = 6.38, *p* = 0.0116; LRT_CTmax = 17.87, *p* = 2.4 × 10⁻⁵), indicating that intraspecific variation is partially structured at the colony level

**Fig. 3:**
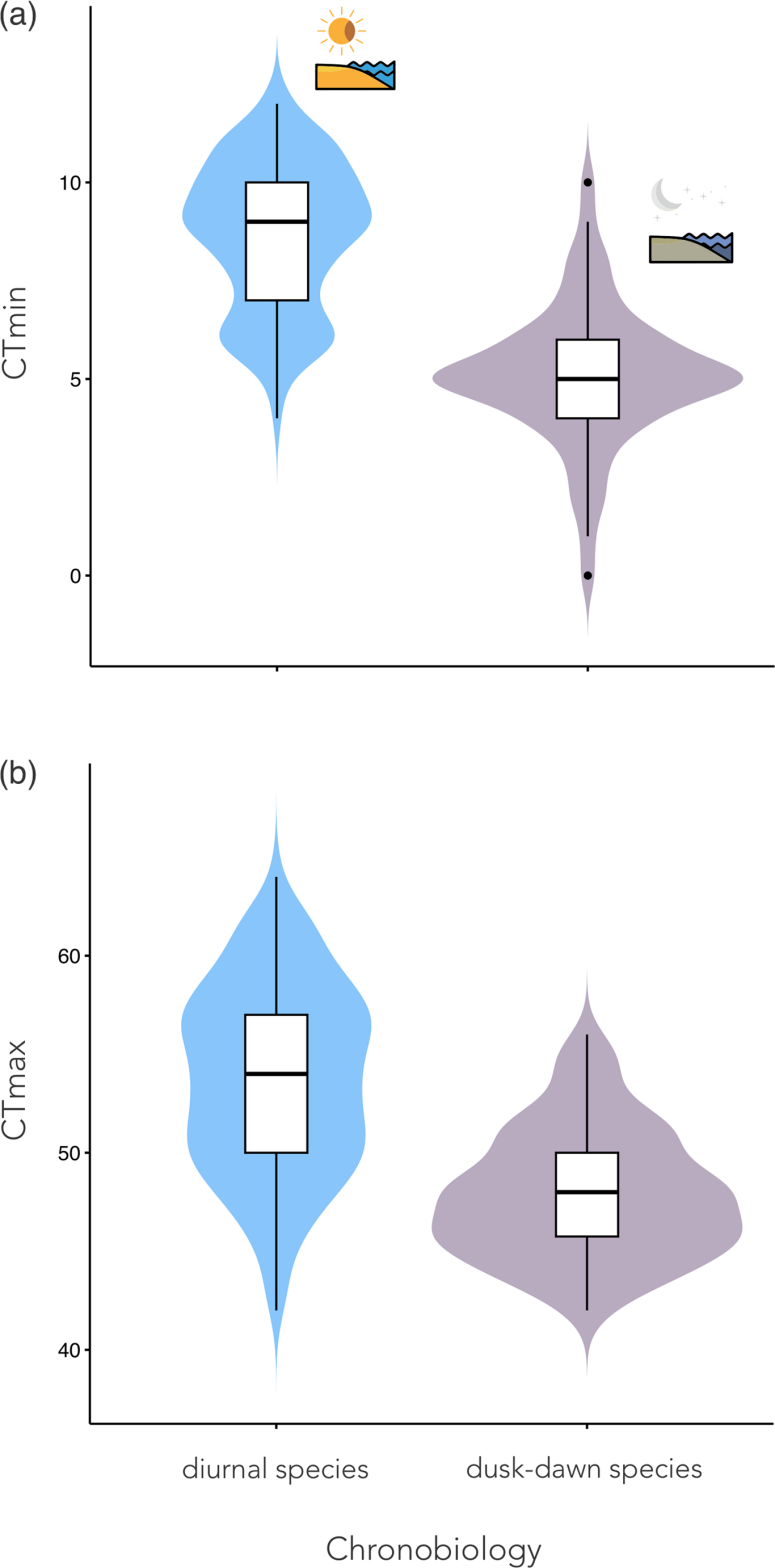
Effect of circadian activity on critical thermal limits (CTmin and CTmax) in *Mycetophylax* ants. Nocturnal individuals (*M. simplex*) exhibited significantly lower CTmin and CTmax compared to diurnal species (*M. morschi* and *M. conformis*) (CTmin: χ² = 113.72, *p* < 2.2 × 10⁻¹⁶; CTmax: χ² = 38.97, *p* < 4.3 × 10⁻¹⁰). Colony effects were also significant (LRT_CTmin = 9.13, *p* = 0.0025; LRT_CTmax = 17.87, *p* = 2.4 × 10⁻⁵), suggesting that thermal tolerance is influenced by both activity rhythm and colony identity

Thermal tolerance thresholds (CTmin and CTmax) were analyzed using linear mixed-effects models, with species identity (Fig. 2) and circadian period (diurnal vs. nocturnal; Fig. 3) as fixed effects, and colony as a random factor to account for non-independence among individuals. For CTmin, the model including circadian period as a fixed effect revealed a significant difference (χ² = 113.72, df = 1, *p* < 2.2 × 10⁻¹⁶; Fig. 3a), with nocturnal individuals exhibiting significantly lower CTmin values (estimate = − 3.57 ± 0.33, *p* < 0.001). The random effect of colony was also significant (LRT = 9.13, *p* = 0.0025). A separate model including species as the fixed effect also showed a strong effect (χ² = 143.94, df = 2, *p* < 2.2 × 10⁻¹⁶; Fig. 2a), with *M. simplex* presenting markedly lower CTmin values compared to the other two congeners. The colony effect remained significant in this model as well (LRT = 6.38, *p* = 0.0116). When comparing the two models, a likelihood ratio test slightly favored the species-only model (χ² = 3.94, df = 1, *p* = 0.047), suggesting that species identity alone may better explain the observed variation in CTmin. However, because species and circadian period are perfectly confounded in these psammophilous *Mycetophylax* ants (i.e., each species is associated exclusively with one circadian condition), the effects of species and circadian rhythms are statistically indistinguishable in this dataset.

For CTmax, circadian rhythms also had a strong effect (χ² = 38.97, df = 1, *p* = 4.3 × 10⁻¹⁰), with nocturnal individuals exhibiting significantly lower CTmax values (estimate = − 6.03 ± 0.97, *p* < 0.001; Fig. 3b). The random effect of colony was again significant (LRT = 17.87, *p* = 2.4 × 10⁻⁵), indicating that colony-level differences contributed to the variability in thermal limits. The model including species as the fixed effect also revealed a significant effect (χ² = 69.72, df = 2, *p* = 7.2 × 10⁻¹⁶; Fig. 2b), with *M. morschi* showing slightly higher CTmax values and *M. simplex* showing significantly lower values compared to *M. conformis*. Model comparison favored the species model over the circadian model (χ² = 6.76, df = 1, *p* = 0.0093), suggesting that species identity better explains the variation in CTmax. Model assumptions were assessed using residual simulations from the DHARMa package, which confirmed that residual distributions and variance structures were within acceptable limits. Overall, these results indicate that species-specific chronobiology plays a key role in determining both lower and upper thermal limits in psammophilous *Mycetophylax*, with additional variability attributable to colony-level effects.

To quantify the contribution of colony-level effects to thermal tolerance, we fitted linear mixed-effects models using colony identity as a random factor. Variance components were extracted to estimate broad-sense heritability (H²), calculated as the proportion of total phenotypic variance attributable to among-colony differences (Table 1). For CTmax, H² was estimated at 0.39, and for CTmin at 0.53. Likelihood ratio tests confirmed that the inclusion of colony as a random effect significantly improved model fit for both traits (CTmax: χ² = 125.63, *p* < 0.001; CTmin: χ² = 189.24, *p* < 0.001). These results suggest a strong genetic or shared environmental component to thermal tolerance, reinforcing the potential for selection to act on colony-level variation in thermal physiology.

**Table 1:** Variance components and broad-sense heritability (H²) estimates for critical thermal limits in *Mycetophylax* ants. Values were obtained using linear mixed-effects models with colony as a random factor. Broad-sense heritability (H²) was calculated as the proportion of total variance attributable to between-colony variance. Likelihood ratio tests (LRT) compare the full model (including colony effect) to a null model without random effects. Both CTmax and CTmin exhibited significant colony-level structure, indicating a genetic or fixed component to thermal tolerance

Trait	Between-colony variance (σ²_colony)	Residual variance (σ²_residual)	Total variance	Heritability (H²)	LRT χ²	*p*-value
CTmax	10.903	16.914	27.817	392	125.63	3.7e-29
CTmin	3.528	3.136	6.664	529	189.24	4.7e-43

## Discussion

Our results reveal pronounced interspecific differences in both CTmin and CTmax among psammophilous *Mycetophylax* species, supporting the idea that critical thermal limits emerge from the interaction of behavioral strategies, physiological constraints, microhabitat exposure, and evolutionary history (Herrando-Pérez et al. 2020; Roeder et al. 2021; Kingsolver and Umbanhowar 2018; Bujan et al. 2020a, b; Bennett et al. 2021). *Mycetophylax simplex* consistently exhibited the lowest thermal thresholds, characterizing it as cold-tolerant but heat-sensitive, while *M. morschi* and *M. conformis* showed higher CTmax values, likely reflecting greater resilience to high-temperature environments. These interspecific differences are consistent with broader evidence that heat tolerance tends to be more ecologically constrained and evolves more slowly than cold tolerance (Bujan et al. 2020a, b; Herrando-Pérez et al. 2020; Bennett et al. 2021), highlighting the potential vulnerability of heat-sensitive species in the face of climate warming. We also detected substantial variation in thermal tolerance among colonies within each species. Our heritability estimates (H² = 0.39 for CTmax and 0.53 for CTmin) indicate that a significant proportion of this variation is genetically or developmentally fixed, and not purely plastic. This contrasts with recent work in *Ectatomma ruidum*, where diel differences in CTmax were observed within colonies, but were not attributable to colony identity or stable individual roles, indicating instead to reversible physiological plasticity (Nelson et al., 2018).

In our system, diel differences in thermal limits are coupled with species-level chronobiological strategies: diurnal species (*M. morschi*, *M. conformis*) consistently showed higher CTmax and broader thermal performance ranges, while *M. simplex*, a twilight and nocturnal forager, exhibited a narrow thermal window and reduced heat tolerance. This strong alignment between thermal physiology and activity rhythm suggests that temporal niche partitioning is not merely behavioral but may be underpinned by heritable traits shaped by natural selection at the colony level. Such evidence highlights a fundamental distinction in how thermal variation can structure insect physiology: through plastic modulation of performance (as in *E. ruidum*) or through genetic differentiation (as in *Mycetophylax*). These findings highlight that plastic and heritable responses to diel and microclimatic variation may operate simultaneously but vary in prevalence across taxa and environmental contexts.

Among the studied species, *M. morschi* displayed the broadest thermal performance range and widest activity window, likely enhancing its capacity to cope with fluctuating thermal conditions. Conversely, *M. simplex* restricted thermal tolerance and narrow temporal niche suggest a heightened vulnerability to thermal extremes. Similar patterns have been observed in other nocturnal ectotherms such as *Megalopta* bees, which exhibit lower CTmax and reduced thermal safety margins compared to their diurnal counterparts (Gonzalez et al. 2023), and nocturnal ants across Mexican forests (Garcia-Robledo et al. 2017). These comparisons reinforce the ecological importance of temporal niche specialization and its role in shaping thermal sensitivity and climate vulnerability.

We found a strong correlation between species-specific circadian rhythms and thermal performance, emphasizing the role of temporal plasticity in ectothermic physiology (Baudier & O’Donnell, 2017; Neptune and Benard 2024). Diurnally active *M. morschi* and *M. conformis*, which routinely forage during peak daytime temperatures, exhibited higher CTmax values and broader thermal performance ranges. In contrast, *M. simplex* restricted its foraging to twilight and nighttime periods, thereby avoiding daytime thermal extremes but increasing its exposure to nocturnal cold stress. Comparable behavioral and physiological patterns have been reported in other ectotherms, such as dune lizards and nocturnal bees, which also show reduced heat tolerance and narrower thermal safety margins (Roeder et al. 2022; Gonzalez et al. 2023; Garcia-Robledo et al. 2017; Herrando-Pérez et al. 2020). These findings support the notion that thermal performance is not fixed, but temporally plastic shaped by diel activity patterns and potentially regulated by internal chronobiological mechanisms (Baudier & O’Donnell, 2017; Neptune and Benard 2024). Importantly, our heritability estimates indicate that variation in CTmax and CTmin is not solely the result of phenotypic plasticity. Broad-sense heritability values of 0.39 for CTmax and 0.53 for CTmin reveal that a substantial portion of this variation is structured at the colony level, suggesting a genetic or developmental basis. Although species identity and circadian rhythm are confounded in our design (each species corresponds to a distinct temporal niche), this overlap highlights the adaptive significance of chronobiology as a dimension of thermal tolerance. This is particularly relevant for ectotherms, in which daily temperature fluctuations impose strong selective pressures (Kingsolver and Umbanhowar 2018).

The relationship between thermal physiology and resource use adds further nuance to the thermal ecology of *Mycetophylax* ants. Although previous studies in seed-harvesting ants have shown that CTmax can be influenced by energy availability and hydration status (Bujan and Kaspari 2017; O’Donnell et al., 2020), such mechanisms may differ in fungus-farming ants like *Mycetophylax*, whose nutrition relies primarily on cultivated fungal symbionts. In coastal dune habitats, nighttime humidity is typically high, likely reducing desiccation risk for *M. simplex* despite its restricted foraging window. However, other traits—particularly body pigmentation—may play a significant role in shaping thermal sensitivity. *M. simplex* has a pale-yellow cuticle, whereas *M. morschi* and *M. conformis* are dark brown. This difference in pigmentation could influence cuticular absorption of solar radiation: lighter-colored individuals tend to reflect more sunlight, potentially reducing body heating under high-exposure conditions, while darker-colored ants absorb more heat, which may be advantageous under cooler or shaded microclimates (Clusella-Trullas et al. 2007; Rajpurohit et al. 2016; Badejo et al. 2020). Beyond thermoregulation, body color may also confer protection against UV radiation and contribute to desiccation resistance (Parkash et al. 2008; Willmer 1982; Law et al. 2020). These combined effects suggest that pigmentation, alongside activity rhythms and habitat use, contributes to interspecific differences in thermal tolerance in coastal *Mycetophylax* species.

Our results also align with recent macroecological findings showing that thermal tolerance in ants is shaped not only by latitude and elevation but also by species-specific traits such as arboreality and chronobiology. Câmara et al. (2024) demonstrated that CTmax exhibits a strong phylogenetic signal, with limited variation along latitudinal gradients but a marked increase with elevation in mid- and high-latitude regions. This pattern is largely attributed to the occurrence of tree-nesting species in thermally heterogeneous canopy environments. In our study, the highest CTmax values were observed in diurnal species occupying more exposed microhabitats, while nocturnal species exhibited significantly lower thermal limits. This diel contrast mirrors patterns of temporal plasticity described in other ectotherms and is consistent with the climatic variability hypothesis (CVH) at fine spatial and temporal scales (Shah et al. 2017, 2020; Sunday et al. 2011; Deutsch et al. 2008). Collectively, our results reinforce the emerging view that the niche asymmetry hypothesis (NAH) may explain large-scale geographic trends in thermal tolerance, whereas CVH better accounts for local variation driven by microclimate and behavioral rhythms (Gaston et al. 2008; Clusella-Trullas et al. 2011).

The use of CTmax as an indicator of vulnerability to thermal stress has been debated, primarily due to its sensitivity to methodological parameters such as ramping rate and endpoint criteria (see Lutterschmidt and Hutchison 1997). However, when standardized appropriately, CTmax has shown strong predictive power for thermal performance and survival under chronic stress. Recent work has directly linked CTmax variation to mortality thresholds in ectothermic species (Cicchino et al. 2023), reinforcing its ecological relevance. In our study, individuals foraging during nocturnal periods consistently exhibited lower CTmax values, reflecting diel modulation of physiological limits. This reduced heat tolerance underlines the limited evolutionary potential for CTmax adjustment, particularly in species already operating near their upper thermal boundaries. Nonetheless, our findings suggest that interspecific and chronobiological differences in CTmax remain ecologically meaningful and should be incorporated into mechanistic models forecasting species responses to ongoing climate warming.

From a conservation standpoint, our results position *Mycetophylax simplex* as an ecological specialist highly vulnerable to both habitat degradation and climate warming. This species is restricted to a narrow range of coastal dunes in southern Brazil, where populations are threatened by anthropogenic pressures such as real estate development, vegetation removal, and dune destabilization. Even modest increases in surface temperature or alterations in soil structure may push colonies beyond their upper thermal limits (CTmax) or below the thresholds required to maintain fungal symbionts, ultimately compromising colony persistence (Lima et al. 2022; McGlynn et al. 2020). Furthermore, the asymmetric evolutionary lability of thermal traits—where CTmin evolves more rapidly than CTmax—suggests that cold-adapted species like *M. simplex* may face reduced adaptive potential underongoing warming trends (Herrando-Pérez et al. 2020; Bennett et al. 2021). The species’ narrow thermal window, as observed in our data, reinforces its heightened susceptibility.

The unique combination of thermal sensitivity, behavioral constraints, and edaphic specialization makes *M. simplex* an ideal sentinel for monitoring the health of restinga ecosystems. Notably, the heritable nature of its thermal tolerance traits suggests limited plasticity in response to rapid environmental change, potentially constraining its adaptive capacity under future climate scenarios. This genetic component reinforces the urgency of conservation measures, as natural selection alone may not operate fast enough to buffer the species against escalating thermal stress. Future research should explore the interactive roles of diet, hydration, and soil microstructure in shaping colony viability, and should also investigate the genetic architecture underlying thermal tolerance, with the aim of informing more targeted and evolutionarily informed conservation strategies.

In conclusion, our findings support the emerging view that physiological metrics such as CTmin and CTmax serve not only as indicators of thermal resilience but also as critical tools for conservation planning. Importantly, we demonstrate that a substantial portion of thermal tolerance variation is structured at the colony level, with broad-sense heritability estimates of 0.39 for CTmax and 0.53 for CTmin. This genetic or developmental component indicates that thermal performance traits in *Mycetophylax* are not purely plastic but may respond to evolutionary pressures, particularly in environments where temperature varies predictably across time. Climate projections for southeastern Brazil predict increasing maximum temperatures, declining precipitation, and extended dry seasons (IPCC 2023). Therefore, the long-term persistence of *Mycetophylax* ants—especially *M. simplex*—depends not only on macrohabitat preservation but also on the maintenance of microenvironmental integrity that buffers thermal extremes. The case of *M. simplex* illustrates how narrow thermal tolerance, restricted foraging behavior, and substrate specialization interact to elevate vulnerability under climate and land-use change. Given its heritable sensitivity to environmental temperature and its ecological specialization, *M. simplex* should be prioritized in conservation actions and recognized as a bioindicator of ecological stability in Brazil’s coastal dune ecosystems.

## Supplementary Information

Below is the link to the electronic supplementary material.

Supplementary Material 1 (DOCX 78.7 KB)

## Data Availability

Data will be made available on request.
