# Supplementary material for: Thermal tolerance and heritability in dune-dwelling ants reveal bioindicator potential for climate vulnerability in coastal ecosystems
Source: Int J Biometeorol. 2026 Feb 6;70(2):51. doi: 10.1007/s00484-025-03081-5 (PMC12881004; doi:10.1007/s00484-025-03081-5)
Supplement: Supplementary file 1 — Supplementary Material 1 (DOCX 78.7 KB) [file 484_2025_3081_MOESM1_ESM.docx]

**Supplementary electronic file**

**Table S1** - Summary statistics for critical thermal minima (CTmin) and maxima (CTmax) in psammophilous *Mycetophylax* ants. Values represent means ± standard deviations (SD), with observed minimum–maximum ranges. Measurements are in degrees Celsius (°C).

| **Species** | **CTmin mean** | **±sd** | **CTmax mean** | **±sd** | **CTmin range** | **CTmax range** |
| --- | --- | --- | --- | --- | --- | --- |
| ***Mycetophylax conformis*** | 9.18 | 1.39 | 52.33 | 4.76 | 6–12 | 43–62 |
| ***Mycetophylax morschi*** | 8.44 | 1.99 | 54.64 | 4.47 | 4–12 | 42–64 |
| ***Mycetophylax simplex*** | 5.09 | 1.72 | 47.84 | 3.19 | 0–10 | 42–56 |

**Table S2** - Critical thermal minima (CTmin) dataset per individuals and colonies.

| **species** | **colony** | **ind** | **type** | **temp** |
| --- | --- | --- | --- | --- |
| **simplex** | MS07 | 13 | noite | 8 |
| **simplex** | MS07 | 14 | noite | 5 |
| **simplex** | MS07 | 15 | noite | 5 |
| **simplex** | MS07 | 16 | noite | 5 |
| **simplex** | MS07 | 17 | noite | 5 |
| **simplex** | MS07 | 18 | noite | 8 |
| **simplex** | MS07 | 19 | noite | 5 |
| **simplex** | MS07 | 20 | noite | 8 |
| **simplex** | MS07 | 21 | noite | 6 |
| **simplex** | MS07 | 22 | noite | 5 |
| **simplex** | MS07 | 23 | noite | 5 |
| **simplex** | MS07 | 24 | noite | 2 |
| **simplex** | MS07 | 43 | noite | 4 |
| **simplex** | MS07 | 44 | noite | 5 |
| **simplex** | MS07 | 45 | noite | 6 |
| **simplex** | MS07 | 46 | noite | 6 |
| **simplex** | MS07 | 47 | noite | 4 |
| **simplex** | MS07 | 48 | noite | 1 |
| **simplex** | MS07 | 49 | noite | 5 |
| **simplex** | MS07 | 50 | noite | 5 |
| **simplex** | MS07 | 51 | noite | 5 |
| **simplex** | MS07 | 52 | noite | 4 |
| **simplex** | MS07 | 53 | noite | 3 |
| **simplex** | MS07 | 54 | noite | 5 |
| **simplex** | MS07 | 55 | noite | 3 |
| **simplex** | MS07 | 56 | noite | 5 |
| **simplex** | MS07 | 57 | noite | 5 |
| **simplex** | MS07 | 58 | noite | 5 |
| **simplex** | MS07 | 59 | noite | 6 |
| **simplex** | MS07 | 60 | noite | 8 |
| **simplex** | MS08 | 1 | noite | 5 |
| **simplex** | MS08 | 2 | noite | 6 |
| **simplex** | MS08 | 3 | noite | 5 |
| **simplex** | MS08 | 4 | noite | 6 |
| **simplex** | MS08 | 5 | noite | 3 |
| **simplex** | MS08 | 6 | noite | 5 |
| **simplex** | MS08 | 7 | noite | 6 |
| **simplex** | MS08 | 38 | noite | 5 |
| **simplex** | MS08 | 39 | noite | 6 |
| **simplex** | MS08 | 40 | noite | 7 |
| **simplex** | MS08 | 41 | noite | 5 |
| **simplex** | MS08 | 42 | noite | 1 |
| **simplex** | MS08 | 43 | noite | 5 |
| **simplex** | MS08 | 44 | noite | 5 |
| **simplex** | MS08 | 45 | noite | 7 |
| **simplex** | MS08 | 46 | noite | 5 |
| **simplex** | MS08 | 47 | noite | 3 |
| **simplex** | MS08 | 48 | noite | 5 |
| **simplex** | MS08 | 49 | noite | 2 |
| **simplex** | MS08 | 50 | noite | 10 |
| **simplex** | MS08 | 51 | noite | 6 |
| **simplex** | MS08 | 52 | noite | 5 |
| **simplex** | MS08 | 53 | noite | 6 |
| **simplex** | MS08 | 54 | noite | 5 |
| **simplex** | MS08 | 55 | noite | 6 |
| **simplex** | MS08 | 56 | noite | 6 |
| **simplex** | MS08 | 57 | noite | 5 |
| **simplex** | MS08 | 58 | noite | 4 |
| **simplex** | MS08 | 59 | noite | 6 |
| **simplex** | MS08 | 60 | noite | 5 |
| **simplex** | MS09 | 13 | noite | 7 |
| **simplex** | MS09 | 14 | noite | 7 |
| **simplex** | MS09 | 15 | noite | 6 |
| **simplex** | MS09 | 16 | noite | 3 |
| **simplex** | MS09 | 17 | noite | 5 |
| **simplex** | MS09 | 18 | noite | 5 |
| **simplex** | MS09 | 19 | noite | 5 |
| **simplex** | MS09 | 20 | noite | 2 |
| **simplex** | MS09 | 21 | noite | 0 |
| **simplex** | MS09 | 22 | noite | 0 |
| **simplex** | MS09 | 23 | noite | 4 |
| **simplex** | MS09 | 24 | noite | 7 |
| **simplex** | MS09 | 43 | noite | 4 |
| **simplex** | MS09 | 44 | noite | 6 |
| **simplex** | MS09 | 45 | noite | 8 |
| **simplex** | MS09 | 46 | noite | 5 |
| **simplex** | MS09 | 47 | noite | 6 |
| **simplex** | MS09 | 48 | noite | 6 |
| **simplex** | MS09 | 49 | noite | 5 |
| **simplex** | MS09 | 50 | noite | 6 |
| **simplex** | MS09 | 51 | noite | 6 |
| **simplex** | MS09 | 52 | noite | 5 |
| **simplex** | MS09 | 53 | noite | 3 |
| **simplex** | MS09 | 54 | noite | 6 |
| **simplex** | MS09 | 55 | noite | 5 |
| **simplex** | MS09 | 56 | noite | 4 |
| **simplex** | MS09 | 57 | noite | 7 |
| **simplex** | MS09 | 58 | noite | 5 |
| **simplex** | MS09 | 59 | noite | 5 |
| **simplex** | MS09 | 60 | noite | 4 |
| **simplex** | MS11 | 13 | noite | 5 |
| **simplex** | MS11 | 14 | noite | 5 |
| **simplex** | MS11 | 15 | noite | 2 |
| **simplex** | MS11 | 16 | noite | 5 |
| **simplex** | MS11 | 17 | noite | 8 |
| **simplex** | MS11 | 18 | noite | 4 |
| **simplex** | MS11 | 19 | noite | 5 |
| **simplex** | MS11 | 20 | noite | 5 |
| **simplex** | MS11 | 21 | noite | 5 |
| **simplex** | MS11 | 22 | noite | 5 |
| **simplex** | MS11 | 23 | noite | 4 |
| **simplex** | MS11 | 24 | noite | 5 |
| **simplex** | MM11 | 43 | noite | 4 |
| **simplex** | MM11 | 44 | noite | 5 |
| **simplex** | MM11 | 45 | noite | 9 |
| **simplex** | MM11 | 46 | noite | 10 |
| **simplex** | MM11 | 47 | noite | 4 |
| **simplex** | MM11 | 48 | noite | 5 |
| **simplex** | MM11 | 49 | noite | 7 |
| **simplex** | MM11 | 50 | noite | 4 |
| **simplex** | MM11 | 51 | noite | 9 |
| **simplex** | MM11 | 52 | noite | 4 |
| **simplex** | MM11 | 53 | noite | 4 |
| **simplex** | MM11 | 54 | noite | 7 |
| **simplex** | MM11 | 55 | noite | 5 |
| **simplex** | MM11 | 56 | noite | 4 |
| **simplex** | MM11 | 57 | noite | 4 |
| **simplex** | MM11 | 58 | noite | 6 |
| **simplex** | MM11 | 59 | noite | 2 |
| **simplex** | MM11 | 60 | noite | 5 |
| **conformis** | MC14 | 13 | dia | 10 |
| **conformis** | MC14 | 14 | dia | 9 |
| **conformis** | MC14 | 15 | dia | 10 |
| **conformis** | MC14 | 16 | dia | 10 |
| **conformis** | MC14 | 17 | dia | 11 |
| **conformis** | MC14 | 18 | dia | 11 |
| **conformis** | MC14 | 19 | dia | 11 |
| **conformis** | MC14 | 20 | dia | 7 |
| **conformis** | MC14 | 21 | dia | 11 |
| **conformis** | MC14 | 22 | dia | 9 |
| **conformis** | MC14 | 23 | dia | 7 |
| **conformis** | MC14 | 24 | dia | 10 |
| **conformis** | MC14 | 29 | dia | 11 |
| **conformis** | MC14 | 30 | dia | 11 |
| **conformis** | MC14 | 31 | dia | 11 |
| **conformis** | MC14 | 32 | dia | 12 |
| **conformis** | MC14 | 47 | dia | 6 |
| **conformis** | MC14 | 48 | dia | 10 |
| **conformis** | MC14 | 49 | dia | 9 |
| **conformis** | MC14 | 50 | dia | 10 |
| **conformis** | MC14 | 51 | dia | 11 |
| **conformis** | MC14 | 52 | dia | 8 |
| **conformis** | MC14 | 53 | dia | 9 |
| **conformis** | MC14 | 54 | dia | 9 |
| **conformis** | MC14 | 55 | dia | 11 |
| **conformis** | MC14 | 56 | dia | 11 |
| **conformis** | MC14 | 57 | dia | 11 |
| **conformis** | MC14 | 58 | dia | 9 |
| **conformis** | MC14 | 59 | dia | 9 |
| **conformis** | MC14 | 60 | dia | 11 |
| **conformis** | MC13 | 13 | dia | 9 |
| **conformis** | MC13 | 14 | dia | 9 |
| **conformis** | MC13 | 15 | dia | 9 |
| **conformis** | MC13 | 16 | dia | 9 |
| **conformis** | MC13 | 17 | dia | 9 |
| **conformis** | MC13 | 18 | dia | 9 |
| **conformis** | MC13 | 19 | dia | 8 |
| **conformis** | MC13 | 20 | dia | 8 |
| **conformis** | MC13 | 21 | dia | 10 |
| **conformis** | MC13 | 22 | dia | 8 |
| **conformis** | MC13 | 23 | dia | 6 |
| **conformis** | MC13 | 24 | dia | 9 |
| **conformis** | MC13 | 37 | dia | 8 |
| **conformis** | MC13 | 38 | dia | 8 |
| **conformis** | MC13 | 39 | dia | 9 |
| **conformis** | MC13 | 40 | dia | 9 |
| **conformis** | MC13 | 41 | dia | 8 |
| **conformis** | MC13 | 42 | dia | 8 |
| **conformis** | MC13 | 43 | dia | 9 |
| **conformis** | MC13 | 44 | dia | 8 |
| **conformis** | MC13 | 45 | dia | 6 |
| **conformis** | MC13 | 46 | dia | 9 |
| **conformis** | MC13 | 47 | dia | 9 |
| **conformis** | MC13 | 48 | dia | 8 |
| **conformis** | MC13 | 59 | dia | 11 |
| **conformis** | MC13 | 60 | dia | 8 |
| **conformis** | MC13 | 61 | dia | 7 |
| **conformis** | MC13 | 62 | dia | 10 |
| **conformis** | MC13 | 63 | dia | 9 |
| **conformis** | MC13 | 64 | dia | 8 |
| **conformis** | MC13 | 65 | dia | 8 |
| **conformis** | MC13 | 66 | dia | 10 |
| **conformis** | MC13 | 67 | dia | 10 |
| **conformis** | MC13 | 68 | dia | 10 |
| **morschi** | MM01 | 11 | dia | 9 |
| **morschi** | MM01 | 12 | dia | 8 |
| **morschi** | MM01 | 13 | dia | 6 |
| **morschi** | MM01 | 14 | dia | 8 |
| **morschi** | MM01 | 15 | dia | 4 |
| **morschi** | MM01 | 16 | dia | 10 |
| **morschi** | MM01 | 17 | dia | 7 |
| **morschi** | MM01 | 18 | dia | 8 |
| **morschi** | MM01 | 19 | dia | 10 |
| **morschi** | MM01 | 20 | dia | 8 |
| **morschi** | MM01 | 41 | dia | 11 |
| **morschi** | MM01 | 42 | dia | 10 |
| **morschi** | MM01 | 43 | dia | 7 |
| **morschi** | MM01 | 44 | dia | 10 |
| **morschi** | MM01 | 45 | dia | 9 |
| **morschi** | MM01 | 46 | dia | 9 |
| **morschi** | MM01 | 47 | dia | 11 |
| **morschi** | MM01 | 48 | dia | 9 |
| **morschi** | MM01 | 49 | dia | 6 |
| **morschi** | MM01 | 50 | dia | 10 |
| **morschi** | MM01 | 51 | dia | 10 |
| **morschi** | MM01 | 52 | dia | 9 |
| **morschi** | MM01 | 53 | dia | 8 |
| **morschi** | MM01 | 54 | dia | 11 |
| **morschi** | MM01 | 55 | dia | 6 |
| **morschi** | MM01 | 56 | dia | 12 |
| **morschi** | MM01 | 57 | dia | 6 |
| **morschi** | MM01 | 58 | dia | 6 |
| **morschi** | MM01 | 59 | dia | 9 |
| **morschi** | MM01 | 60 | dia | 10 |
| **morschi** | MM02 | 11 | dia | 9 |
| **morschi** | MM02 | 12 | dia | 10 |
| **morschi** | MM02 | 13 | dia | 10 |
| **morschi** | MM02 | 14 | dia | 9 |
| **morschi** | MM02 | 15 | dia | 11 |
| **morschi** | MM02 | 16 | dia | 8 |
| **morschi** | MM02 | 17 | dia | 11 |
| **morschi** | MM02 | 18 | dia | 8 |
| **morschi** | MM02 | 19 | dia | 10 |
| **morschi** | MM02 | 20 | dia | 11 |
| **morschi** | MM02 | 41 | dia | 7 |
| **morschi** | MM02 | 42 | dia | 10 |
| **morschi** | MM02 | 43 | dia | 7 |
| **morschi** | MM02 | 44 | dia | 10 |
| **morschi** | MM02 | 45 | dia | 7 |
| **morschi** | MM02 | 46 | dia | 11 |
| **morschi** | MM02 | 47 | dia | 9 |
| **morschi** | MM02 | 48 | dia | 12 |
| **morschi** | MM02 | 49 | dia | 7 |
| **morschi** | MM02 | 50 | dia | 9 |
| **morschi** | MM02 | 51 | dia | 7 |
| **morschi** | MM02 | 52 | dia | 7 |
| **morschi** | MM02 | 53 | dia | 5 |
| **morschi** | MM02 | 54 | dia | 10 |
| **morschi** | MM02 | 55 | dia | 9 |
| **morschi** | MM02 | 56 | dia | 10 |
| **morschi** | MM02 | 57 | dia | 11 |
| **morschi** | MM02 | 58 | dia | 11 |
| **morschi** | MM02 | 59 | dia | 5 |
| **morschi** | MM02 | 60 | dia | 7 |
| **morschi** | MM03 | 11 | dia | 8 |
| **morschi** | MM03 | 12 | dia | 11 |
| **morschi** | MM03 | 13 | dia | 6 |
| **morschi** | MM03 | 14 | dia | 7 |
| **morschi** | MM03 | 15 | dia | 10 |
| **morschi** | MM03 | 16 | dia | 10 |
| **morschi** | MM03 | 17 | dia | 11 |
| **morschi** | MM03 | 18 | dia | 7 |
| **morschi** | MM03 | 19 | dia | 12 |
| **morschi** | MM03 | 20 | dia | 10 |
| **morschi** | MM03 | 41 | dia | 6 |
| **morschi** | MM03 | 42 | dia | 10 |
| **morschi** | MM03 | 43 | dia | 11 |
| **morschi** | MM03 | 44 | dia | 11 |
| **morschi** | MM03 | 45 | dia | 6 |
| **morschi** | MM03 | 46 | dia | 6 |
| **morschi** | MM03 | 47 | dia | 6 |
| **morschi** | MM03 | 48 | dia | 9 |
| **morschi** | MM03 | 49 | dia | 9 |
| **morschi** | MM03 | 50 | dia | 9 |
| **morschi** | MM03 | 51 | dia | 6 |
| **morschi** | MM03 | 52 | dia | 9 |
| **morschi** | MM03 | 53 | dia | 8 |
| **morschi** | MM03 | 54 | dia | 10 |
| **morschi** | MM03 | 55 | dia | 10 |
| **morschi** | MM03 | 56 | dia | 8 |
| **morschi** | MM03 | 57 | dia | 9 |
| **morschi** | MM03 | 58 | dia | 9 |
| **morschi** | MM03 | 59 | dia | 12 |
| **morschi** | MM03 | 60 | dia | 8 |
| **morschi** | MM04 | 11 | dia | 7 |
| **morschi** | MM04 | 12 | dia | 8 |
| **morschi** | MM04 | 13 | dia | 10 |
| **morschi** | MM04 | 14 | dia | 8 |
| **morschi** | MM04 | 15 | dia | 7 |
| **morschi** | MM04 | 16 | dia | 9 |
| **morschi** | MM04 | 17 | dia | 11 |
| **morschi** | MM04 | 18 | dia | 11 |
| **morschi** | MM04 | 19 | dia | 5 |
| **morschi** | MM04 | 20 | dia | 6 |
| **morschi** | MM04 | 41 | dia | 6 |
| **morschi** | MM04 | 42 | dia | 6 |
| **morschi** | MM04 | 43 | dia | 6 |
| **morschi** | MM04 | 44 | dia | 6 |
| **morschi** | MM04 | 45 | dia | 6 |
| **morschi** | MM04 | 46 | dia | 6 |
| **morschi** | MM04 | 47 | dia | 9 |
| **morschi** | MM04 | 48 | dia | 6 |
| **morschi** | MM04 | 49 | dia | 6 |
| **morschi** | MM04 | 50 | dia | 12 |
| **morschi** | MM04 | 51 | dia | 11 |
| **morschi** | MM04 | 52 | dia | 9 |
| **morschi** | MM04 | 53 | dia | 6 |
| **morschi** | MM04 | 54 | dia | 6 |
| **morschi** | MM04 | 55 | dia | 6 |
| **morschi** | MM04 | 56 | dia | 11 |
| **morschi** | MM04 | 57 | dia | 6 |
| **morschi** | MM04 | 58 | dia | 6 |
| **morschi** | MM04 | 59 | dia | 6 |
| **morschi** | MM04 | 60 | dia | 9 |
| **morschi** | MM06 | 11 | dia | 9 |
| **morschi** | MM06 | 12 | dia | 6 |
| **morschi** | MM06 | 13 | dia | 10 |
| **morschi** | MM06 | 14 | dia | 9 |
| **morschi** | MM06 | 15 | dia | 9 |
| **morschi** | MM06 | 16 | dia | 9 |
| **morschi** | MM06 | 17 | dia | 6 |
| **morschi** | MM06 | 18 | dia | 10 |
| **morschi** | MM06 | 19 | dia | 6 |
| **morschi** | MM06 | 20 | dia | 6 |
| **morschi** | MM06 | 41 | dia | 7 |
| **morschi** | MM06 | 42 | dia | 10 |
| **morschi** | MM06 | 43 | dia | 10 |
| **morschi** | MM06 | 44 | dia | 9 |
| **morschi** | MM06 | 45 | dia | 5 |
| **morschi** | MM06 | 46 | dia | 9 |
| **morschi** | MM06 | 47 | dia | 9 |
| **morschi** | MM06 | 48 | dia | 12 |
| **morschi** | MM06 | 49 | dia | 9 |
| **morschi** | MM06 | 50 | dia | 10 |
| **morschi** | MM06 | 51 | dia | 9 |
| **morschi** | MM06 | 52 | dia | 4 |
| **morschi** | MM06 | 53 | dia | 10 |
| **morschi** | MM06 | 54 | dia | 10 |
| **morschi** | MM06 | 55 | dia | 6 |
| **morschi** | MM06 | 56 | dia | 9 |
| **morschi** | MM06 | 57 | dia | 11 |
| **morschi** | MM06 | 58 | dia | 10 |
| **morschi** | MM06 | 59 | dia | 6 |
| **morschi** | MM06 | 60 | dia | 5 |

**Table S3**- Critical thermal maxima (CTmax) dataset per individuals and colonies.

| **specie** | **colony** | **ind** | **type** | **temp** |
| --- | --- | --- | --- | --- |
| **simplex** | MS07 | 1 | noite | 50 |
| **simplex** | MS07 | 2 | noite | 50 |
| **simplex** | MS07 | 3 | noite | 47 |
| **simplex** | MS07 | 4 | noite | 54 |
| **simplex** | MS07 | 5 | noite | 46 |
| **simplex** | MS07 | 6 | noite | 49 |
| **simplex** | MS07 | 7 | noite | 53 |
| **simplex** | MS07 | 8 | noite | 55 |
| **simplex** | MS07 | 9 | noite | 44 |
| **simplex** | MS07 | 10 | noite | 44 |
| **simplex** | MS07 | 11 | noite | 47 |
| **simplex** | MS07 | 12 | noite | 46 |
| **simplex** | MS07 | 25 | noite | 50 |
| **simplex** | MS07 | 26 | noite | 53 |
| **simplex** | MS07 | 27 | noite | 46 |
| **simplex** | MS07 | 28 | noite | 51 |
| **simplex** | MS07 | 29 | noite | 49 |
| **simplex** | MS07 | 30 | noite | 46 |
| **simplex** | MS07 | 31 | noite | 48 |
| **simplex** | MS07 | 32 | noite | 52 |
| **simplex** | MS07 | 33 | noite | 48 |
| **simplex** | MS07 | 34 | noite | 49 |
| **simplex** | MS07 | 35 | noite | 51 |
| **simplex** | MS07 | 36 | noite | 48 |
| **simplex** | MS07 | 37 | noite | 54 |
| **simplex** | MS07 | 38 | noite | 49 |
| **simplex** | MS07 | 39 | noite | 48 |
| **simplex** | MS07 | 40 | noite | 46 |
| **simplex** | MS07 | 41 | noite | 47 |
| **simplex** | MS07 | 42 | noite | 56 |
| **simplex** | MS08 | 8 | noite | 52 |
| **simplex** | MS08 | 9 | noite | 44 |
| **simplex** | MS08 | 10 | noite | 49 |
| **simplex** | MS08 | 11 | noite | 46 |
| **simplex** | MS08 | 12 | noite | 47 |
| **simplex** | MS08 | 13 | noite | 46 |
| **simplex** | MS08 | 14 | noite | 47 |
| **simplex** | MS08 | 15 | noite | 47 |
| **simplex** | MS08 | 16 | noite | 45 |
| **simplex** | MS08 | 17 | noite | 45 |
| **simplex** | MS08 | 18 | noite | 43 |
| **simplex** | MS08 | 19 | noite | 43 |
| **simplex** | MS08 | 20 | noite | 44 |
| **simplex** | MS08 | 21 | noite | 45 |
| **simplex** | MS08 | 22 | noite | 42 |
| **simplex** | MS08 | 23 | noite | 43 |
| **simplex** | MS08 | 24 | noite | 43 |
| **simplex** | MS08 | 25 | noite | 46 |
| **simplex** | MS08 | 26 | noite | 42 |
| **simplex** | MS08 | 27 | noite | 47 |
| **simplex** | MS08 | 28 | noite | 45 |
| **simplex** | MS08 | 29 | noite | 46 |
| **simplex** | MS08 | 30 | noite | 44 |
| **simplex** | MS08 | 31 | noite | 45 |
| **simplex** | MS08 | 32 | noite | 48 |
| **simplex** | MS08 | 33 | noite | 48 |
| **simplex** | MS08 | 34 | noite | 46 |
| **simplex** | MS08 | 35 | noite | 45 |
| **simplex** | MS08 | 36 | noite | 46 |
| **simplex** | MS08 | 37 | noite | 47 |
| **simplex** | MS09 | 1 | noite | 49 |
| **simplex** | MS09 | 2 | noite | 49 |
| **simplex** | MS09 | 3 | noite | 44 |
| **simplex** | MS09 | 4 | noite | 48 |
| **simplex** | MS09 | 5 | noite | 51 |
| **simplex** | MS09 | 6 | noite | 54 |
| **simplex** | MS09 | 7 | noite | 46 |
| **simplex** | MS09 | 8 | noite | 48 |
| **simplex** | MS09 | 9 | noite | 52 |
| **simplex** | MS09 | 10 | noite | 52 |
| **simplex** | MS09 | 11 | noite | 49 |
| **simplex** | MS09 | 12 | noite | 51 |
| **simplex** | MS09 | 25 | noite | 45 |
| **simplex** | MS09 | 26 | noite | 50 |
| **simplex** | MS09 | 27 | noite | 51 |
| **simplex** | MS09 | 28 | noite | 46 |
| **simplex** | MS09 | 29 | noite | 48 |
| **simplex** | MS09 | 30 | noite | 48 |
| **simplex** | MS09 | 31 | noite | 51 |
| **simplex** | MS09 | 32 | noite | 55 |
| **simplex** | MS09 | 33 | noite | 49 |
| **simplex** | MS09 | 34 | noite | 46 |
| **simplex** | MS09 | 35 | noite | 44 |
| **simplex** | MS09 | 36 | noite | 51 |
| **simplex** | MS09 | 37 | noite | 48 |
| **simplex** | MS09 | 38 | noite | 43 |
| **simplex** | MS09 | 39 | noite | 48 |
| **simplex** | MS09 | 40 | noite | 48 |
| **simplex** | MS09 | 41 | noite | 46 |
| **simplex** | MS09 | 42 | noite | 51 |
| **simplex** | MS11 | 1 | noite | 55 |
| **simplex** | MS11 | 2 | noite | 48 |
| **simplex** | MS11 | 3 | noite | 55 |
| **simplex** | MS11 | 4 | noite | 51 |
| **simplex** | MS11 | 5 | noite | 45 |
| **simplex** | MS11 | 6 | noite | 52 |
| **simplex** | MS11 | 7 | noite | 52 |
| **simplex** | MS11 | 8 | noite | 46 |
| **simplex** | MS11 | 9 | noite | 45 |
| **simplex** | MS11 | 10 | noite | 46 |
| **simplex** | MS11 | 11 | noite | 45 |
| **simplex** | MS11 | 12 | noite | 51 |
| **simplex** | MS11 | 25 | noite | 49 |
| **simplex** | MS11 | 26 | noite | 44 |
| **simplex** | MS11 | 27 | noite | 46 |
| **simplex** | MS11 | 28 | noite | 51 |
| **simplex** | MS11 | 29 | noite | 50 |
| **simplex** | MS11 | 30 | noite | 47 |
| **simplex** | MS11 | 31 | noite | 48 |
| **simplex** | MS11 | 32 | noite | 44 |
| **simplex** | MS11 | 33 | noite | 44 |
| **simplex** | MS11 | 34 | noite | 45 |
| **simplex** | MS11 | 35 | noite | 48 |
| **simplex** | MS11 | 36 | noite | 51 |
| **simplex** | MS11 | 37 | noite | 48 |
| **simplex** | MS11 | 38 | noite | 46 |
| **simplex** | MS11 | 39 | noite | 45 |
| **simplex** | MS11 | 40 | noite | 48 |
| **simplex** | MS11 | 41 | noite | 44 |
| **simplex** | MS11 | 42 | noite | 49 |
| **conformis** | MC14 | 1 | dia | 55 |
| **conformis** | MC14 | 2 | dia | 48 |
| **conformis** | MC14 | 3 | dia | 49 |
| **conformis** | MC14 | 4 | dia | 62 |
| **conformis** | MC14 | 5 | dia | 51 |
| **conformis** | MC14 | 6 | dia | 57 |
| **conformis** | MC14 | 7 | dia | 48 |
| **conformis** | MC14 | 8 | dia | 48 |
| **conformis** | MC14 | 9 | dia | 56 |
| **conformis** | MC14 | 10 | dia | 57 |
| **conformis** | MC14 | 11 | dia | 56 |
| **conformis** | MC14 | 12 | dia | 62 |
| **conformis** | MC14 | 25 | dia | 44 |
| **conformis** | MC14 | 26 | dia | 43 |
| **conformis** | MC14 | 27 | dia | 53 |
| **conformis** | MC14 | 28 | dia | 47 |
| **conformis** | MC14 | 33 | dia | 43 |
| **conformis** | MC14 | 34 | dia | 43 |
| **conformis** | MC14 | 35 | dia | 50 |
| **conformis** | MC14 | 36 | dia | 52 |
| **conformis** | MC14 | 37 | dia | 56 |
| **conformis** | MC14 | 38 | dia | 52 |
| **conformis** | MC14 | 39 | dia | 50 |
| **conformis** | MC14 | 40 | dia | 47 |
| **conformis** | MC14 | 41 | dia | 50 |
| **conformis** | MC14 | 42 | dia | 50 |
| **conformis** | MC14 | 43 | dia | 43 |
| **conformis** | MC14 | 44 | dia | 59 |
| **conformis** | MC14 | 45 | dia | 59 |
| **conformis** | MC14 | 46 | dia | 47 |
| **conformis** | MC13 | 1 | dia | 59 |
| **conformis** | MC13 | 2 | dia | 50 |
| **conformis** | MC13 | 3 | dia | 54 |
| **conformis** | MC13 | 4 | dia | 55 |
| **conformis** | MC13 | 5 | dia | 54 |
| **conformis** | MC13 | 6 | dia | 58 |
| **conformis** | MC13 | 7 | dia | 58 |
| **conformis** | MC13 | 8 | dia | 61 |
| **conformis** | MC13 | 9 | dia | 48 |
| **conformis** | MC13 | 10 | dia | 49 |
| **conformis** | MC13 | 11 | dia | 58 |
| **conformis** | MC13 | 12 | dia | 52 |
| **conformis** | MC13 | 25 | dia | 57 |
| **conformis** | MC13 | 26 | dia | 46 |
| **conformis** | MC13 | 27 | dia | 56 |
| **conformis** | MC13 | 28 | dia | 52 |
| **conformis** | MC13 | 29 | dia | 58 |
| **conformis** | MC13 | 30 | dia | 47 |
| **conformis** | MC13 | 31 | dia | 56 |
| **conformis** | MC13 | 32 | dia | 56 |
| **conformis** | MC13 | 33 | dia | 52 |
| **conformis** | MC13 | 34 | dia | 52 |
| **conformis** | MC13 | 35 | dia | 59 |
| **conformis** | MC13 | 36 | dia | 49 |
| **conformis** | MC13 | 49 | dia | 50 |
| **conformis** | MC13 | 50 | dia | 53 |
| **conformis** | MC13 | 51 | dia | 49 |
| **conformis** | MC13 | 52 | dia | 46 |
| **conformis** | MC13 | 53 | dia | 53 |
| **conformis** | MC13 | 54 | dia | 53 |
| **conformis** | MC13 | 55 | dia | 53 |
| **conformis** | MC13 | 56 | dia | 50 |
| **conformis** | MC13 | 57 | dia | 48 |
| **conformis** | MC13 | 58 | dia | 45 |
| **conformis** | MC13 | 1 | dia | 59 |
| **conformis** | MC13 | 2 | dia | 50 |
| **conformis** | MC13 | 3 | dia | 54 |
| **conformis** | MC13 | 4 | dia | 55 |
| **conformis** | MC13 | 5 | dia | 54 |
| **conformis** | MC13 | 6 | dia | 58 |
| **conformis** | MC13 | 7 | dia | 58 |
| **conformis** | MC13 | 8 | dia | 61 |
| **conformis** | MC13 | 9 | dia | 48 |
| **conformis** | MC13 | 10 | dia | 49 |
| **conformis** | MC13 | 11 | dia | 58 |
| **conformis** | MC13 | 12 | dia | 52 |
| **conformis** | MC13 | 25 | dia | 57 |
| **conformis** | MC13 | 26 | dia | 46 |
| **conformis** | MC13 | 27 | dia | 56 |
| **conformis** | MC13 | 28 | dia | 52 |
| **conformis** | MC13 | 29 | dia | 58 |
| **conformis** | MC13 | 30 | dia | 47 |
| **conformis** | MC13 | 31 | dia | 56 |
| **conformis** | MC13 | 32 | dia | 56 |
| **conformis** | MC13 | 33 | dia | 52 |
| **conformis** | MC13 | 34 | dia | 52 |
| **conformis** | MC13 | 35 | dia | 59 |
| **conformis** | MC13 | 36 | dia | 49 |
| **conformis** | MC13 | 49 | dia | 50 |
| **conformis** | MC13 | 50 | dia | 53 |
| **conformis** | MC13 | 51 | dia | 49 |
| **conformis** | MC13 | 52 | dia | 46 |
| **conformis** | MC13 | 53 | dia | 53 |
| **conformis** | MC13 | 54 | dia | 53 |
| **conformis** | MC13 | 55 | dia | 53 |
| **conformis** | MC13 | 56 | dia | 50 |
| **conformis** | MC13 | 57 | dia | 48 |
| **conformis** | MC13 | 58 | dia | 45 |
| **morschi** | MM01 | 1 | dia | 52 |
| **morschi** | MM01 | 2 | dia | 58 |
| **morschi** | MM01 | 3 | dia | 49 |
| **morschi** | MM01 | 4 | dia | 50 |
| **morschi** | MM01 | 5 | dia | 49 |
| **morschi** | MM01 | 6 | dia | 50 |
| **morschi** | MM01 | 7 | dia | 55 |
| **morschi** | MM01 | 8 | dia | 52 |
| **morschi** | MM01 | 9 | dia | 52 |
| **morschi** | MM01 | 10 | dia | 55 |
| **morschi** | MM01 | 21 | dia | 46 |
| **morschi** | MM01 | 22 | dia | 62 |
| **morschi** | MM01 | 23 | dia | 62 |
| **morschi** | MM01 | 24 | dia | 55 |
| **morschi** | MM01 | 25 | dia | 59 |
| **morschi** | MM01 | 26 | dia | 51 |
| **morschi** | MM01 | 27 | dia | 52 |
| **morschi** | MM01 | 28 | dia | 60 |
| **morschi** | MM01 | 29 | dia | 48 |
| **morschi** | MM01 | 30 | dia | 57 |
| **morschi** | MM01 | 31 | dia | 55 |
| **morschi** | MM01 | 32 | dia | 58 |
| **morschi** | MM01 | 33 | dia | 57 |
| **morschi** | MM01 | 34 | dia | 62 |
| **morschi** | MM01 | 35 | dia | 59 |
| **morschi** | MM01 | 36 | dia | 48 |
| **morschi** | MM01 | 37 | dia | 54 |
| **morschi** | MM01 | 38 | dia | 52 |
| **morschi** | MM01 | 39 | dia | 48 |
| **morschi** | MM01 | 40 | dia | 58 |
| **morschi** | MM02 | 1 | dia | 51 |
| **morschi** | MM02 | 2 | dia | 56 |
| **morschi** | MM02 | 3 | dia | 61 |
| **morschi** | MM02 | 4 | dia | 54 |
| **morschi** | MM02 | 5 | dia | 51 |
| **morschi** | MM02 | 6 | dia | 59 |
| **morschi** | MM02 | 7 | dia | 51 |
| **morschi** | MM02 | 8 | dia | 51 |
| **morschi** | MM02 | 9 | dia | 51 |
| **morschi** | MM02 | 10 | dia | 56 |
| **morschi** | MM02 | 21 | dia | 60 |
| **morschi** | MM02 | 22 | dia | 50 |
| **morschi** | MM02 | 23 | dia | 46 |
| **morschi** | MM02 | 24 | dia | 56 |
| **morschi** | MM02 | 25 | dia | 56 |
| **morschi** | MM02 | 26 | dia | 64 |
| **morschi** | MM02 | 27 | dia | 56 |
| **morschi** | MM02 | 28 | dia | 60 |
| **morschi** | MM02 | 29 | dia | 56 |
| **morschi** | MM02 | 30 | dia | 60 |
| **morschi** | MM02 | 31 | dia | 56 |
| **morschi** | MM02 | 32 | dia | 57 |
| **morschi** | MM02 | 33 | dia | 52 |
| **morschi** | MM02 | 34 | dia | 56 |
| **morschi** | MM02 | 35 | dia | 56 |
| **morschi** | MM02 | 36 | dia | 52 |
| **morschi** | MM02 | 37 | dia | 46 |
| **morschi** | MM02 | 38 | dia | 62 |
| **morschi** | MM02 | 39 | dia | 50 |
| **morschi** | MM02 | 40 | dia | 56 |
| **morschi** | MM03 | 1 | dia | 59 |
| **morschi** | MM03 | 2 | dia | 54 |
| **morschi** | MM03 | 3 | dia | 54 |
| **morschi** | MM03 | 4 | dia | 57 |
| **morschi** | MM03 | 5 | dia | 54 |
| **morschi** | MM03 | 6 | dia | 54 |
| **morschi** | MM03 | 7 | dia | 54 |
| **morschi** | MM03 | 8 | dia | 57 |
| **morschi** | MM03 | 9 | dia | 54 |
| **morschi** | MM03 | 10 | dia | 49 |
| **morschi** | MM03 | 21 | dia | 59 |
| **morschi** | MM03 | 22 | dia | 51 |
| **morschi** | MM03 | 23 | dia | 59 |
| **morschi** | MM03 | 24 | dia | 59 |
| **morschi** | MM03 | 25 | dia | 42 |
| **morschi** | MM03 | 26 | dia | 59 |
| **morschi** | MM03 | 27 | dia | 57 |
| **morschi** | MM03 | 28 | dia | 49 |
| **morschi** | MM03 | 29 | dia | 50 |
| **morschi** | MM03 | 30 | dia | 50 |
| **morschi** | MM03 | 31 | dia | 49 |
| **morschi** | MM03 | 32 | dia | 56 |
| **morschi** | MM03 | 33 | dia | 59 |
| **morschi** | MM03 | 34 | dia | 59 |
| **morschi** | MM03 | 35 | dia | 53 |
| **morschi** | MM03 | 36 | dia | 48 |
| **morschi** | MM03 | 37 | dia | 54 |
| **morschi** | MM03 | 38 | dia | 50 |
| **morschi** | MM03 | 39 | dia | 50 |
| **morschi** | MM03 | 40 | dia | 50 |
| **morschi** | MM04 | 1 | dia | 61 |
| **morschi** | MM04 | 2 | dia | 58 |
| **morschi** | MM04 | 3 | dia | 61 |
| **morschi** | MM04 | 4 | dia | 61 |
| **morschi** | MM04 | 5 | dia | 50 |
| **morschi** | MM04 | 6 | dia | 56 |
| **morschi** | MM04 | 7 | dia | 50 |
| **morschi** | MM04 | 8 | dia | 54 |
| **morschi** | MM04 | 9 | dia | 56 |
| **morschi** | MM04 | 10 | dia | 45 |
| **morschi** | MM04 | 21 | dia | 61 |
| **morschi** | MM04 | 22 | dia | 48 |
| **morschi** | MM04 | 23 | dia | 54 |
| **morschi** | MM04 | 24 | dia | 51 |
| **morschi** | MM04 | 25 | dia | 62 |
| **morschi** | MM04 | 26 | dia | 57 |
| **morschi** | MM04 | 27 | dia | 51 |
| **morschi** | MM04 | 28 | dia | 61 |
| **morschi** | MM04 | 29 | dia | 48 |
| **morschi** | MM04 | 30 | dia | 57 |
| **morschi** | MM04 | 31 | dia | 64 |
| **morschi** | MM04 | 32 | dia | 61 |
| **morschi** | MM04 | 33 | dia | 52 |
| **morschi** | MM04 | 34 | dia | 57 |
| **morschi** | MM04 | 35 | dia | 51 |
| **morschi** | MM04 | 36 | dia | 55 |
| **morschi** | MM04 | 37 | dia | 57 |
| **morschi** | MM04 | 38 | dia | 59 |
| **morschi** | MM04 | 39 | dia | 57 |
| **morschi** | MM04 | 40 | dia | 57 |
| **morschi** | MM06 | 1 | dia | 58 |
| **morschi** | MM06 | 2 | dia | 52 |
| **morschi** | MM06 | 3 | dia | 57 |
| **morschi** | MM06 | 4 | dia | 47 |
| **morschi** | MM06 | 5 | dia | 55 |
| **morschi** | MM06 | 6 | dia | 51 |
| **morschi** | MM06 | 7 | dia | 61 |
| **morschi** | MM06 | 8 | dia | 61 |
| **morschi** | MM06 | 9 | dia | 62 |
| **morschi** | MM06 | 10 | dia | 51 |
| **morschi** | MM06 | 21 | dia | 54 |
| **morschi** | MM06 | 22 | dia | 57 |
| **morschi** | MM06 | 23 | dia | 59 |
| **morschi** | MM06 | 24 | dia | 54 |
| **morschi** | MM06 | 25 | dia | 54 |
| **morschi** | MM06 | 26 | dia | 54 |
| **morschi** | MM06 | 27 | dia | 48 |
| **morschi** | MM06 | 28 | dia | 57 |
| **morschi** | MM06 | 29 | dia | 49 |
| **morschi** | MM06 | 30 | dia | 57 |
| **morschi** | MM06 | 31 | dia | 54 |
| **morschi** | MM06 | 32 | dia | 62 |
| **morschi** | MM06 | 33 | dia | 51 |
| **morschi** | MM06 | 34 | dia | 57 |
| **morschi** | MM06 | 35 | dia | 54 |
| **morschi** | MM06 | 36 | dia | 54 |
| **morschi** | MM06 | 37 | dia | 50 |
| **morschi** | MM06 | 38 | dia | 52 |
| **morschi** | MM06 | 39 | dia | 57 |
| **morschi** | MM06 | 40 | dia | 54 |
